# Short-Term Low-Temperature Storage and Cryopreservation of Qihe Crucian Carp (*Carassius auratus*) Sperm

**DOI:** 10.3390/ani15050698

**Published:** 2025-02-27

**Authors:** Xi Shi, Jiayin Xu, Yujie Hou, Zhen Wei, Lufang Guo, Xiao Ma, Limin Wu, Wenge Ma, Xue Tian, Khor Waiho, Xuejun Li

**Affiliations:** 1 College of Life Sciences, Henan Normal University, Xinxiang 453007, China; shixi@htu.edu.cn; 2 College of Fisheries, Henan Normal University, Xinxiang 453007, China; xjy_2025@163.com (J.X.); 13623736705@139.com (Y.H.); 18238650981@163.com (Z.W.); 18438891802@163.com (L.G.); maxiao@htu.edu.cn (X.M.); wulimin@htu.edu.cn (L.W.); mawenge@htu.edu.cn (W.M.); tianxue@htu.edu.cn (X.T.); 3 Hangzhou Xiaoshan Donghai Breeding Co., Ltd., Hangzhou 311200, China; 4 Higher Institution Centre of Excellence (HICoE), Institute of Tropical Aquaculture and Fisheries, Universiti Malaysia Terengganu, Kuala Nerus 21030, Terengganu, Malaysia; waiho@umt.edu.my; 5 Observation and Research Station on Water Ecosystem in Danjiangkou Reservoir of Henan Province, Nanyang 474450, China

**Keywords:** sperm storage, cryopreservation, Qihe crucian carp, motility, cryoprotectant

## Abstract

Qihe crucian carp *Carassius auratus* is one of the major economic aquacultural fish species in China. Due to environmental degradation and the impact of the reproduction mode of gynogenesis, the wild population of Qihe crucian carp exhibits an extremely low proportion of males. Therefore, it is imperative to develop the sperm preservation technology for Qihe crucian carp. In this study, the indicators, including sperm motility, sperm movement time, and sperm lifetime, were applied to evaluate the preservation effects of various extenders, cryoprotectant types and concentrations, and preservation manners, thus establishing the techniques of sperm short-term storage and cryopreservation for Qihe crucian carp. The results showed that HBSS was the most suitable extender solution, in which the sperm motility reached 93.00%. Under room temperature (26 °C), the sperm motility showed no significant differences across six different cryopreservation solutions. Under low temperature (4 °C), a 15% MeOH solution presented the most effective preservation effects of sperm in the short-term low-temperature storage. For cryopreservation in liquid nitrogen, the sperm activity in 20% DMSO was significantly higher than that in other solutions, representing the optimal cryopreservation solution. Furthermore, it was found that the sperm activity preserved by liquid nitrogen was significantly higher than that preserved by the ultra-freezer (−80 °C), indicating that the ultra-freezer cannot substitute for liquid nitrogen in cryopreservation. This is the first study to investigate the preservation of Qihe crucian carp sperm, providing valuable technical support for both genetic resource conservation and artificial breeding programs.

## Simple Summary

The sperm characteristics are various among fish species, which leads to different methods of sperm preservation for specific species. The sperm preservation technique for Qihe crucian carp, one of the major economic aquacultural fish species in China, has not been reported previously. This study aimed to construct the method of sperm preservation for Qihe crucian carp. The results showed that Hank’s Balanced Salt Solution (HBSS) was the best extender solution, and 15% MeOH and 20% DMSO were the optimal cryopreservation solutions for low-temperature storage and liquid nitrogen preservation, respectively. Further, under current conditions, an ultra-freezer (−80 °C) could not replace liquid nitrogen for sperm cryopreservation in Qihe crucian carp. This is the first study to investigate the preservation of Qihe crucian carp sperm, which will provide valuable technical support for both genetic resource conservation and artificial breeding programs.

## 1. Introduction

Crucian carp (*Carassius auratus*) is one of the most important economic freshwater fish, which has been widely farmed in China owing to its advantages of fast growth rate, palatability, high nutritional values, and strong resistance to environments [1,2]. According to the China Fishery Statistical Yearbook, the aquaculture output of crucian carp in 2022 reached more than 2.849 million tons, ranking fourth among the major freshwater aquaculture species in China. Qihe crucian carp is a special local variation population and mainly distributed in the Qihe River Basin [3]. It is well known as “double-backed crucian carp” because its body is much thicker than other crucian carp species in morphology [4]. Qihe crucian carp is traditionally cultured in the north of Henan Province, China, which has already been a lucrative aquaculture species for local fishermen [3,5].

In recent years, the wild genetic resources of Qihe crucian carp have been threatened by many aspects, such as intense environmental pollution, construction of engineering facilities, overfishing, and insufficient protection efforts [6,7]. As a result, the wild population of Qihe crucian carp has been sharply declining, and the ecological areas suitable for its reproduction and survival have been gradually shrinking [8]. Nowadays, the wild population of Qihe crucian carp has reached an endangered level [7]. Additionally, the threat of genetic pollution from other farmed crucian carp species has also arisen, so the protection of its germplasm resources has attracted much attention from researchers [6]. As a consequence, Qihe crucian carp has already been listed as one of the “List of Henan Key Protected Wild Animals” in 2018 by the Henan Provincial Government. Therefore, construction of the preservation technology of Qihe crucian carp sperm will be helpful to establish a sperm bank to protect its genetic resources and maintain its genetic diversity [9,10].

Generally, Qihe crucian carp have two reproductive modes, namely, gynogenesis and sexual reproduction [11,12]. Gynogenesis has become the main reproductive mode of Qihe crucian carp in practice due to the scarcity of male individuals, resulting in the phenomenon of “nine females of ten fish” in the natural population. If this trend continues, the male individuals will decline gradually over time or even disappear someday. Further, it has been found that the offspring produced by sexual reproduction showed obvious advantages in growth performance and feed utilization compared with those produced by gynogenesis in Qihe crucian carp [3]. Thus, preserving the sperm and conducting artificial sexual reproduction when required not only helps to solve the gender imbalance problem in the Qihe crucian carp population but also benefits the aquaculture and increases the economic returns of the aquaculturists [3,9,13].

Fish sperm preservation can be divided into short-term preservation and long-term cryopreservation [14,15]. In the short-term preservation, the fish sperm are usually preserved at room temperature or at low temperatures (close to 0 °C), and sperm activity can generally be maintained for several days or weeks, which can be used for short-term artificial breeding [15,16]. Of course, if long-term preservation of sperm activity is desired, the fish sperm should be preserved under ultra-low temperatures, such as liquid nitrogen or −80 °C [9,13]. The cryopreservation of fish sperm involves the dilution in a suitable extender solution and the addition of the optimal cryoprotectant, which can avoid freezing damage and allow them in a stable ultra-low temperature environment [16]. For most freshwater fish, sperm is immediately activated when it encounters fresh water or hypotonic solutions [17]. In order to improve sperm storability, the extender solution should include nutrients, have a good buffering capacity, and be isotonic with semen, which can provide a suitable environment for sperm and effectively prevent sperm from being activated [14,18]. Additionally, the extender solution should be non-toxic or have minimal toxicity to sperm, and the composition should be as simple as possible. In an ultra-low temperature environment, the sperm cells are dormant, and cell metabolism has almost completely stopped, thus achieving the goal of long-term preservation. When needed, the sperm can be taken out and thawed with an appropriate method, which can restore normal physiological functions and be used for artificial insemination [9,14,15].

The sperm of some protected fish or quality varieties can be preserved by using sperm cryopreservation technology to avoid the decline of fish germplasm resources caused by habitat pollution, overfishing, and long-term inbreeding [9]. Additionally, the fish sperm cryopreservation technology is also beneficial to provide continuous materials for biotechnology research [19]. Moreover, the sperm cryopreservation can solve many practical problems in fish breeding, such as the asynchronous maturation of males and females, sex reversal, and difficulty in mating due to geographical isolation [9,14]. Since Blaxter first preserved the sperm of herring (*Clupea harengus*) in 1953 [20], plenty of sperm storage techniques have been successfully constructed in fish. As an important economic fish group, the sperm storage methods of some cyprinids have been developed, such as common carp *Cyprinus carpio* [21], grass carp *Ctenopharyngodon idella* [22], goldfish *C. auratus* [23], Naleh fish *Barbonymus* sp. [24], mirror carp *C. carpio* [25], etc. However, up to now, no studies on the sperm preservation of Qihe crucian carp have been reported. Therefore, this study aims to screen the optimal extender, cryoprotectant, and cryopreservation manner for the preservation of Qihe crucian carp sperm, thus providing technical support for its conservation of genetic resources and artificial breeding.

## 2. Materials and Methods

### 2.1. Experimental Fish and the Collection of Sperm

Male Qihe crucian carp (n = 20), having well-developed gonads, were selected as experimental fish, with a mean body weight of (238.79 ± 15.08) g and a mean body length of (25.90 ± 0.44) cm. Before sperm collection, the experimental fish were cultured in a tank for 14-day acclimation, keeping them in a greenhouse with a good aquacultural condition. The aquacultural temperature was kept between 22 and 26 °C. The feed used was a commercial fish feed with a protein content of 34%. The tank was continuously aerated to keep the saturated condition of dissolved oxygen. The parameters of water quality were daily monitored by a YSI multi-parameter water quality detector in the morning. The water quality parameters were as follows: dissolved oxygen ≥ 5.0 mg/L; pH 7.6–8.3; and total ammonia-nitrogen < 0.1 mg/L. Afterwards, the experimental fish were artificially induced into spawning by injection with a mixture of luteinizing hormone-releasing hormone (LHRH-A2) and domperidone (DOM) in dosages of 3 μg/kg and 1 mg/kg (according to the instructions of the manufacturer), respectively. The experimental fish were anesthetized with MS-222 solution (100 mg/L) 12 h after the administration of the hormone and the sperm were collected by abdominal pressure. During the sperm collection, the abdomen of the experimental fish must be kept dry to avoid sperm contamination by urine, feces, and water. Additionally, sperm should avoid direct sun exposure during collection. At last, the collected sperm were transferred to 2.0 mL cryogenic vials (Corning Inc., New York, NY, USA) for the following analysis.

### 2.2. Sperm Activity Measurement

A drop of the sperm was placed on a dry slide to make a smear. The sperm activity was measured under a microscope (400×) after the activation by distilled water. The evaluation of sperm activity included sperm motility, sperm movement time, and sperm lifetime.

Sperm motility: the percentage of motile sperm after sperm activation;

Sperm movement time: the time from sperm activation to 90% of sperm shaking in place and not demonstrating forward motion;

Sperm lifetime: the time from sperm activation to 90% of sperm stopping motion.

Only samples with sperm motility higher than 90% could be used for subsequent cryopreservation experiments. In this study, all sperm activity was tested for three replicates, and each replicate was performed three times.

### 2.3. Screening of the Optimal Extender Solution

Four commonly used sperm preservation extenders for aquatic animals were selected, including Ringer’s Solution (RS), Hank’s Balanced Salt Solution (HBSS), Common Carp Sperm Extender 2 (CCSE2), and D-17 diluent (D-17), and their compositions were shown in Table 1. The sperm of the experimental fish were diluted at the ratio of 1:6 with these four extenders, respectively. The dilution ratio was determined based on the pre-experiment. The mixture was gently shaken. The diluted sperm were placed on a dry slide to make a smear, and then the sperm activity was measured according to the method previously described. The optimal extender solution was screened according to the sperm activity.

### 2.4. Screening of the Optimal Cryopreservation Solution

Four commonly used sperm cryoprotectants for aquatic animals, including glycerol, dimethyl sulfoxide (DMSO), methanol (MeOH), and ethylene glycol (EG), were selected. In the pre-experiment, the concentration of cryoprotectants was all first set to 15%, and it was found that DMSO and MeOH showed better preservation effects, so another two groups were added for these two cryoprotectants with the concentration of 20% [29]. Finally, six different types of cryopreservation solutions were set up with the optimal extender solution obtained above as the solvent.

Firstly, whether the negative effects of cryopreservation solutions on sperm activity existed at room temperature (26 °C) was tested. The sperm of the experimental fish was mixed with each cryopreservation solution at the ratio of 1:6, respectively. Then the sperm activity was measured as previously described. Secondly, screening the optimal cryopreservation solution for short-term storage under low temperature (4 °C) [15]. Six cryopreservation solutions were tested as before; however, the difference was that after mixing the cryopreservation solution and the sperm, the mixture was placed at 4 °C for 2 h, and then the sperm activity was detected. Finally, screening the optimal cryopreservation solution for cryopreservation at liquid nitrogen. The fresh sperm was mixed with each cryopreservation solution at the ratio of 1:6, respectively. Then the cryogenic vial with the sperm mixture was pre-cooled at 4 °C for 5 min, balanced at 7 cm above liquid nitrogen for 10 min, and then immersed in liquid nitrogen for cryopreservation, following the methodology described by Lichtenstein et al. (2010) [30] with slight modifications. After 24 h, take the sperm mixture out from liquid nitrogen and quickly thaw it in a water bath with a constant temperature of 37 °C for 50 s [30], and then continue to thaw at room temperature. Afterwords, the sperm activity was measured as previously described.

### 2.5. Choosing the Cryopreservation Manner

In order to further test whether ultra-low temperature storage at −80 °C can replace liquid nitrogen storage, the sperm activity after ultra-freezer (−80 °C) storage was studied. Similarly, two cryopreservation solutions (15% DMSO and 20% DMSO) showing better preservation effects in liquid nitrogen were selected to be mixed with the fresh sperm, and the ratio of sperm and cryopreservation solution was 1:6. After mixing, the cryogenic vial with the sperm mixture was pre-cooled at 4 °C for 5 min and then transferred to an ultra-freezer (−80 °C) for storage. After 24 h, the sperm mixture was thawed, and the sperm activity was detected as previously described.

### 2.6. Statistical Analysis

Statistical analysis was performed using SPSS 21.0 for Windows (SPSS, Michigan Avenue, Chicago, IL, USA). Data were presented as mean ± standard deviation (S.D.). The significant differences were tested by one-way ANOVA and Tukey’s multiple range test, and the differences were considered significant at *p* < 0.05 and extremely significant at *p* < 0.01.

## 3. Results

### 3.1. The Optimal Extender Solution

**Figure 1:**
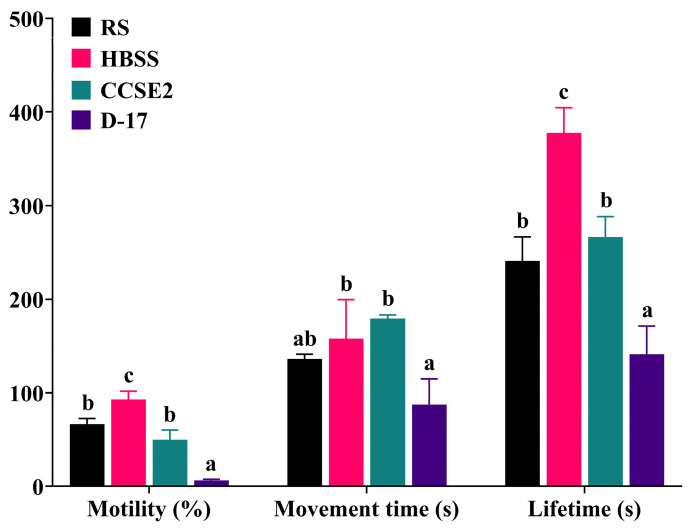
Effects of different extender solutions on sperm activity of Qihe crucian carp. RS: Ringer’s Solution; HBSS: Hank’s Balanced Salt Solution; CCSE2: Common Carp Sperm Extender 2; D-17: D-17 diluent. Values are means ± S.D. of three replicates, and columns with different letters indicate the significant differences (*p* < 0.05).

The effects of four extender solutions on sperm activities were shown in Figure 1. The sperm motility and sperm lifetime of the HBSS group were (93.00 ± 5.00) % and (377.67 ± 15.68) s, which were significantly higher and longer than those of the other three groups (*p* < 0.05). The sperm movement time of the HBSS group was (158.00 ± 24.03) s, which was significantly longer than that of the D-17 group but showed no significant differences with the CCSE2 and RS groups. In summary, the HBSS group exhibited higher sperm activity, and, consequently, HBSS was considered the optimal extender solution.

### 3.2. Effects of Different Cryopreservation Solutions on Sperm Activities at Room Temperature

**Figure 2:**
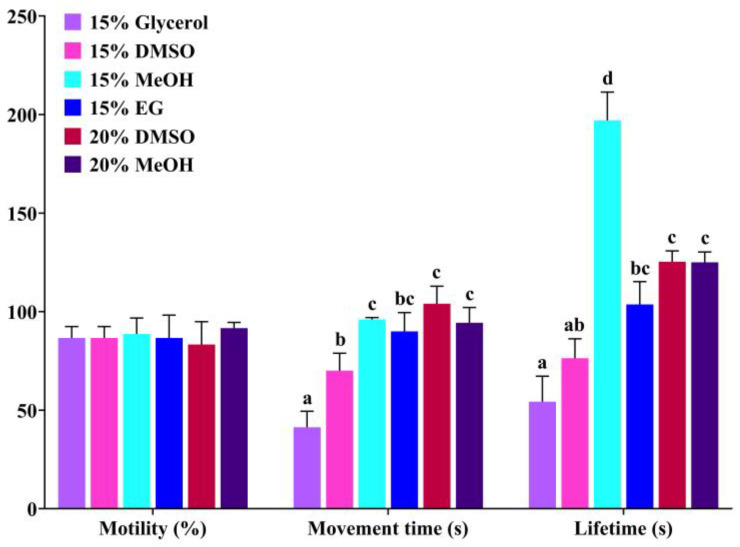
Effects of different cryopreservation solutions on sperm activity of Qihe crucian carp at a room temperature of 26 °C. Values are means ± S.D. of three replicates, and columns with different letters indicate the significant differences (*p* < 0.05).

The effects of six cryopreservation solutions on sperm activities at room temperature (26 °C) were shown in Figure 2. The results showed that the sperm motility presented no significant difference among different treatments (*p* > 0.05). For movement time, the 15% MeOH, 20% DMSO, and 20% MeOH groups exhibited the longest sperm movement time, which was significantly higher than that of the 15% glycerol group and 15% DMSO group (*p* < 0.05), but they showed no significant difference with that of the 15% EG group (*p* > 0.05). Similarly, the 15% MeOH group showed the longest sperm lifetime, followed by the 20% DMSO and 20% MeOH groups, and the 15% glycerol group displayed the shortest sperm lifetime. In conclusion, the negative effects of 15% MeOH, 20% DMSO, and 20% MeOH on sperm activity were minimal under room temperature conditions, and they could be used as candidate cryopreservation solutions.

### 3.3. The Optimal Cryopreservation Solution for Short-Term Storage of Sperm at Low Temperature

**Figure 3:**
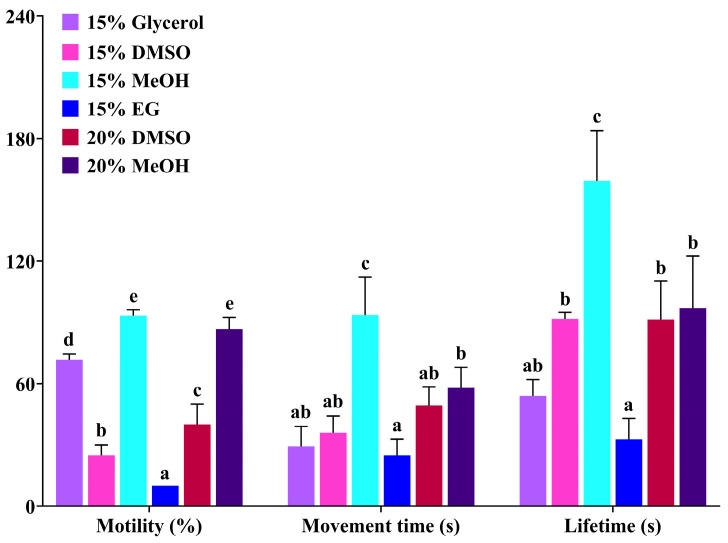
Effects of different cryopreservation solutions on sperm activity of Qihe crucian carp for short-term storage at 4 °C. Values are means ± S.D. of three replicates, and columns with different letters indicate the significant differences (*p* < 0.05).

In order to identify the optimum protocol for short-term storage of sperm at low temperature, six types of cryopreservation solutions were tested at 4 °C (Figure 3). The results showed that the sperm motility of the 15% MeOH and 20% MeOH groups was highest, which was significantly higher than that of other groups (*p* < 0.05). Additionally, the sperm movement time and sperm lifetime of the 15% MeOH group were the longest, which were significantly longer than those of the other five groups (*p* < 0.05). Therefore, 15% MeOH was the optimal cryopreservation solution for short-term storage at low temperature.

### 3.4. The Optimal Cryopreservation Solution and Manner for Sperm Cryopreservation

**Figure 4:**
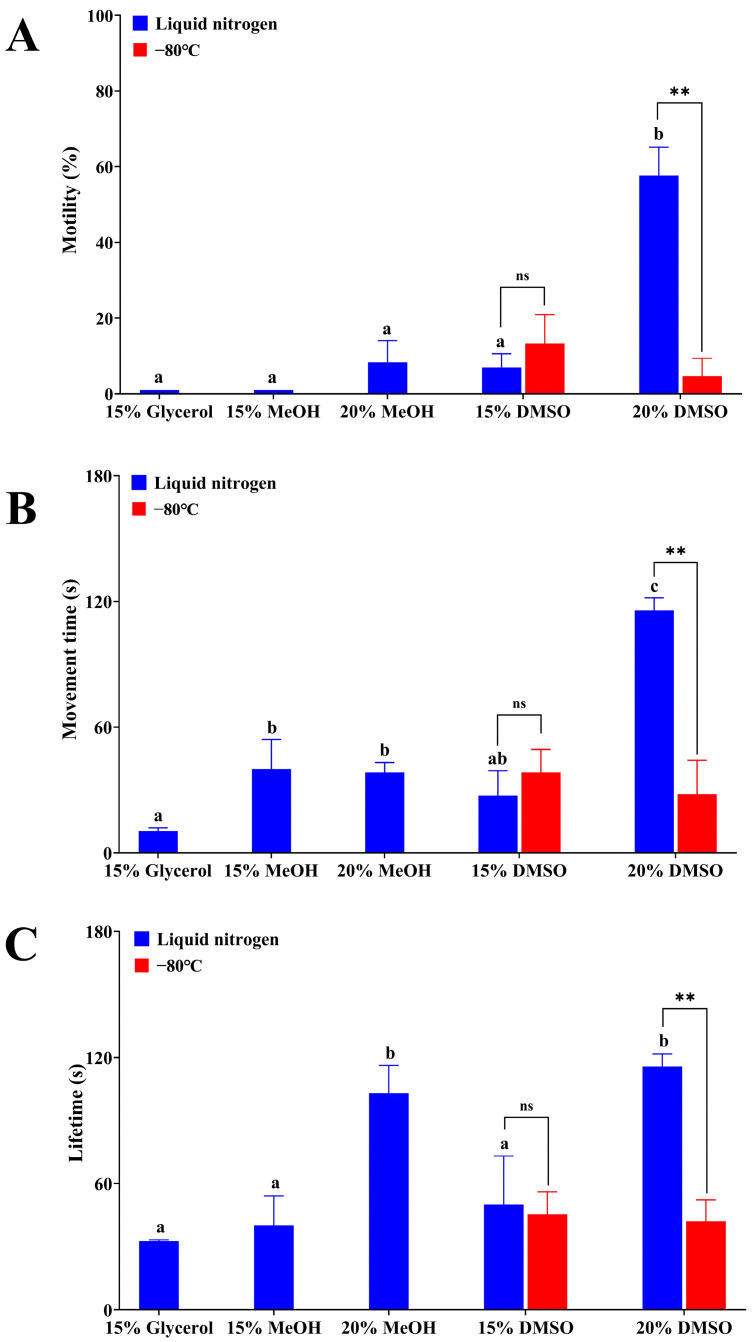
Effects of different cryopreservation solutions and preservation manners on sperm activity of Qihe crucian carp for cryopreservation. (**A**) Sperm motility; (**B**) Sperm movement time; (**C**) Sperm lifetime. Values are means ± S.D. of three replicates, and columns with different letters indicated the significant differences among various cryopreservation solutions (*p* < 0.05), and the asterisks above the columns indicated significant differences between two preservation manners by using the same cryopreservation solution (** *p* < 0.01). ns: not significant (*p* > 0.05).

At first, the effects of cryopreservation solutions on sperm activity under liquid nitrogen conditions were tested. The results showed that in the 15% EG group, all sperm died after freezing in liquid nitrogen and had no sperm motility. The preservative effects of other cryopreservation solutions were shown in Figure 4. The sperm motility was significantly higher, and the sperm movement time was significantly longer in the 20% DMSO group than those in the other groups (*p* < 0.05). In addition, the 20% DMSO group and the 20% MeOH group exhibited significantly longer sperm lifetime than other groups (*p* < 0.05). As a consequence, the 20% DMSO was the optimal cryopreservation solution for sperm cryopreservation under liquid nitrogen conditions.

Moreover, in order to test whether an ultra-freezer (−80 °C) can replace liquid nitrogen in Qihe crucian carp sperm cryopreservation, two cryopreservation solutions of 15% DMSO and 20% DMSO were studied (Figure 4). The results showed that in 15% DMSO solution, the sperm activity including motility, movement time and lifetime presented no significant differences between liquid nitrogen group and ultra-freezer (−80 °C) group, however, they were both relatively lower when compared with that in the 20% DMSO solution. On the other hand, in the 20% DMSO solution, the sperm motility was significantly higher, and the sperm movement time and lifetime were significantly longer in the liquid nitrogen group than those in the ultra-freezer (−80 °C) group (*p* < 0.05), indicating that liquid nitrogen preservation was the better cryopreservation manner than ultra-freezer (−80 °C) preservation.

## 4. Discussion

In practice, no unified standard for fish sperm extender solutions has been identified at present, and the types and concentrations of sperm extenders should be selected based on the characteristics of sperm from different fish species [18,19]. Several extender solutions have been successfully used for fish sperm preservation, such as NaCl [31], HBSS [27], RS [26], CCSE1-CCSE6 [21], D-17 [28], and so on. For example, in Mekong catfish *Pangasius bocourti*, modified HBSS was the most suitable extender [32]; while in pejerrey *Odontesthes bonariensis*, two extenders presented no significant differences on sperm motility indexes [30]. In this study, four commonly used fish sperm extender solutions were selected. The results showed that the D-17 group showed the lowest sperm activity, indicating that D-17 had adverse effects on the sperm motility of Qihe crucian carp; however, D-17 showed the highest post-thaw sperm motility in *Varicorhinus barbatulus* [28]. The different influences may be explained by the species-specific biological characteristics of sperm in various fish [14]. This result further highlights the importance of screening the optimal extender before fish sperm preservation. In this study, the HBSS group showed the highest sperm motility and the longest sperm lifetime, demonstrating that HBSS is the most suitable extender solution for Qihe crucian carp, which can provide a more suitable survival environment and nutrition for sperm.

In low or ultra-low temperature environments, appropriate cryoprotectants are needed to protect the sperm cells from damage during freezing and thawing [9,13,17]. Different cryoprotectants have varying effects on the activity of fish sperm, and the effectiveness of cryoprotectants was species-specific [9,18]. Additionally, studies indicated that most of the cryoprotectants were toxic to sperm cells, and the higher the concentration, the more toxic [13,16]. Therefore, screening the suitable cryoprotectant and determining the optimal concentration were the focus of sperm cryopreservation [26]. Some cryoprotectants, including EG, glycerol, DMSO, MeOH, dimethyl acetamide (DMAC), have been successfully applied for fish sperm cryopreservation [9,19,33]. Among these cryoprotectants, MeOH and DMSO are widely chosen and applied in various marine and freshwater fish species [9,18,34]. In this study, six types of commonly used cryopreservation solutions were composed using different cryoprotectants and concentrations [13]. At room temperature, the adverse impacts of various cryopreservation solutions on sperm activity were found to be minimal, indicating that the toxicity of these solutions on Qihe crucian carp sperm was relatively low. Furthermore, at low temperature (4 °C), 15% MeOH demonstrated superior preservation effects of sperm activity, making it the most suitable short-term low-temperature preservation solution for Qihe crucian carp. Similarly, using MeOH as a cryoprotectant yielded favorable results in short-term low-temperature sperm preservation for European eel *A. anguilla* [15] and coppernose bluegill (CBG) *Lepomis macrochirus purpurescens* [35].

In liquid nitrogen, the preservation effects of different cryopreservation solutions on Qihe crucian carp sperm were varied from those at low temperature. The 20% DMSO solution exhibited the best performances in sperm protection after liquid nitrogen cryopreservation. In fact, DMSO has long been regarded as the preferred cryoprotectant in most studies, which is probably because of its rapid penetration into the cells and its interaction with the phospholipids at the sperm membrane [18,33,34]. For example, in stone flounder *Kareius bicoloratus*, DMSO effectively preserves sperm activity; however, its optimal concentration in the cryoprotectant solution depends on the type of diluents used [36]. Similarly, in some cyprinids, DMSO performed well in antifreezing in sperm cryopreservation [21,24,37]. Further, compared to other concentrations of DMSO, 10% DMSO exhibited superior protective effects for red-spotted grouper *Epinephelus akaara* sperm [38] and goldfish sperm [39]. In theory, the higher concentrations of cryoprotectants offer better protective effects for sperm; nevertheless, some studies reported that excessively high cryoprotectant levels are toxic to sperm cells [13]. Moreover, due to the obvious physiological differences in semen among various fish species, it is crucial to assess the effectiveness and determine the optimal concentration of cryoprotectants before sperm cryopreservation. Some studies suggested that high concentrations of DMSO are toxic to fish sperm [34]. In this study, 15% DMSO and 20% DMSO were applied, and the results showed that compared with 15% DMSO, 20% DMSO presented significantly better protective effects on sperm, indicating that Qihe crucian carp may have stronger resistance to DMSO. Of course, in other studies, 20% DMSO has been applied several times [29,40].

Normally, the cryopreservation of fish sperm is frozen in liquid nitrogen. However, some studies have tried to use ultra-freezers to preserve sperm in aquatic animals [41,42]. Compared with the liquid nitrogen cryopreservation, ultra-freezer (−80 °C) preservation is simpler, more controllable, more effective, has greater storage capacity, and is lower cost [43]. In the present study, the preservation effects of ultra-freezer (−80 °C) and liquid nitrogen cryopreservation on sperm of Qihe crucian carp were compared. The results showed that in 20% DMSO cryopreservation solution, the sperm activity of liquid nitrogen storage was significantly higher than that of ultra-freezer (−80 °C) storage, and the sperm motility in ultra-freezer (−80 °C) storage was quite low, indicating that using ultra-freezer (−80 °C) could not be as an alternative to the use of liquid nitrogen in the sperm preservation of Qihe crucian carp. However, in brown-marbled grouper *E. fuscoguttatus* and streaked prochilod *Prochilodus lineatus*, the researchers demonstrated that an −80 °C ultra-freezer can effectively substitute liquid nitrogen for sperm freezing in these two species [42,43]. The discrepancies in various studies may be attributed to the differences in the sperm physiological characteristics of specific species, the compositions of extenders and cryopreservation solutions, etc. Generally, sperm cryopreservation can cause damage to cells by the ice crystal formed during the cooling and freezing process, which ultimately affects plasma membrane, mitochondria, and chromatin structure [9]. When the storage temperature is lower than −132 °C, the cell aqueous solution is in a glass-like state. However, when the storage temperature is approximately −80 °C, it will form larger ice crystals, which may cause irreversible damage to the cytoskeleton and other structures within the cell and negatively influence the sperm activity [41,44]. This may explain the significantly reduced sperm activity at −80 °C compared to that in liquid nitrogen in the present study.

As an important freshwater economic fish in China, crucian carp has been extensively farmed [3,45]. The sperm preservation technology of crucian carp has also been widely studied. However, different conclusions were obtained among various studies. For instance, Zeng et al. (2024) showed that the sperm cryopreservation method for three kinds of crucian carp was various, while D-20 with 10% DMSO was best for red crucian carp (*C. auratus* red var., RCC), and D-14 as a diluent and 15% DMSO as a cryoprotectant was best for white crucian carp (*C. cuvieri*, WCC) and hybrid crucian carp (WCC♀ × RCC♂) [45]. Additionally, recent studies reported that 10% DMSO combined with 15% egg yolk and 20 min pre-freezing was the best treatment for sperm cryopreservation of *C. auratus* [46,47]. Taghizadeh et al. (2013) concluded that the Goldfish Sperm Extender 2 (GFSE 2) with 10% DMSO can keep the utmost mobility and the motility duration of sperm of *C. auratus gibelio* [39]. In theory, different crucian carp species or populations show different sperm qualities and characteristics [48,49], which highlights the importance of the study on the sperm preservation method in a specific population. For Qihe crucian carp, HBSS with 20% DMSO can keep the highest sperm activity in liquid nitrogen. This study firstly reported the preservation method of Qihe crucian carp sperm, which will facilitate its breeding and aquaculture.

## 5. Conclusions

The protocols of short-term low-temperature storage and cryopreservation of Qihe crucian carp sperm were studied. The results showed that HBSS was the best extender solution and could preserve sperm activity well. At low temperature (4 °C), 15% MeOH could be regarded as the best cryopreservation solution. In terms of cryopreservation, 20% DMSO was the optimal cryopreservation solution for liquid nitrogen preservation. However, under current conditions, an ultra-freezer (−80 °C) could not replace liquid nitrogen for sperm cryopreservation in Qihe crucian carp. The sperm preservation technology established in this study provided strong support for the conservation of genetic resources, biological research, and artificial breeding in Qihe crucian carp.

## Figures and Tables

**Table 1:** Compositions of the extender solutions used in the experiment.

Extender Solution	Compositions (mM)
RS ^1^	111.11 mM NaCl, 1.87 mM KCl, 1.08 mM CaCl_2_, 2.38 mM NaHCO_3_, 0.07 mM NaH_2_PO_4_, 11.11 mM glucose
HBSS ^2^	134.97 mM NaCl, 5.28 mM KCl, 0.79 mM MgSO_4_·7H_2_O, 0.20 mM Na_2_HPO_4_·12H_2_O, 0.40 mM KH_2_PO_4_, 4.11 mM NaHCO_3_, 5.50 mM glucose
CCSE2 ^3^	58.58 mM NaCl, 100.33 mM sucrose
D-17 ^4^	153.85 mM NaCl, 6.67 mM KCl, 83.33 mM glucose

All reagents above were purchased from Aladdin Holdings Group Co., Ltd., (Beijing, China). ^1^ RS: Ringer’s Solution [26]; ^2^ HBSS: Hank’s Balanced Salt Solution [27]; ^3^ CCSE2: Common Carp Sperm Extender 2 [21]; ^4^ D-17: D-17 diluent [28].

## Data Availability

The data presented in this study are available on request from the corresponding author.
